# Efficacy of perifracture site injections for pain control following distal radius and ankle fracture surgery: a meta-analysis of randomized controlled trials

**DOI:** 10.1186/s13018-026-07072-z

**Published:** 2026-07-17

**Authors:** Byung-Ho Yoon, Minsub Kim, Young Hak Roh

**Affiliations:** Department of Orthopaedic Surgery, Ewha Womans University Medical Center, Ewha Womans University College of Medicine, 1071 Anyangcheon-ro, Yangcheon-gu, Seoul, 07985 South Korea

**Keywords:** Injection, Postoperative pain, Distal extremity, Fracture surgery, Randomized controlled trial, Meta-analysis

## Abstract

**Background:**

Surgical site analgesic injections are used as part of multimodal analgesia in elective orthopedic surgery, but their effectiveness in orthopedic trauma surgery, particularly for distal extremity fractures, remains controversial. This meta-analysis aimed to assess the efficacy of peri-fracture site local analgesic injections on postoperative pain and opioid consumption after distal radius and ankle fracture surgery.

**Methods:**

A comprehensive search of PubMed, the Cochrane Library, and EMBASE was performed to identify randomized controlled trials assessing the efficacy of perifracture site injections for pain control after distal extremity fracture surgery. Pooled analyses compared postoperative pain intensity and total opioid consumption between the injection and control groups.

**Results:**

Five randomized controlled trials comprising 390 participants were included. The pooled analysis showed that the injection group had significantly lower pain scores at 4 h postoperatively (SMD − 0.34; 95% CI − 0.56 to − 0.12; I^2^ = 37%). However, no significant differences in pain scores were observed between 8 and 48 h, with substantial heterogeneity at 8 and 24 h (8 h: SMD − 0.18; 95% CI − 0.78 to 0.42, I^2^ = 86%; 24 h: SMD − 0.23; 95% CI − 0.81 to 0.35, I^2^ = 85%). Total opioid consumption was significantly lower in the injection group (SMD − 0.29; 95% CI − 0.52 to − 0.07; I^2^ = 6%).

**Conclusions:**

Peri-fracture site injection for distal radius and ankle fractures provided significant analgesic efficacy confined to the early postoperative period, along with an overall reduction in opioid consumption. Further investigations are needed to establish optimal injection protocols and to determine whether this approach can provide more sustained postoperative pain relief. *Registry number*: CRD420251185330.

## Background

Postoperative pain is a critical concern following fracture surgery, as it not only reduces patient satisfaction but also delays the initiation of rehabilitation, and compromises functional recovery [1, 2]. Despite its clinical importance, postoperative pain after fracture surgery is often underestimated or inadequately managed, partly because it is perceived as an unavoidable consequence of trauma and surgical intervention [2, 3]. Multimodal pain management strategies are used in several musculoskeletal surgeries to address this challenge, incorporating the systemic administration of oral or intravenous analgesics and opioids, regional or peripheral nerve blocks, and local anesthetic techniques such as wound infiltration or intra-articular injection [1, 4–7].

Peri-surgical site local analgesic injections have been utilized in elective procedures, such as arthroplasty or sports medicine surgery [6, 8, 9]. This approach provides localized analgesia by blocking pain conduction at the peripheral site of infiltration, while offering several advantages over systemic opioid administration and nerve blocks. It preserves motor function, reduces opioid-related adverse effects, and is relatively simple and safe compared to nerve blocks [10]. Several studies evaluating local anesthetic infiltration for postoperative pain control have demonstrated satisfactory efficacy, showing improvements in overall pain scores and patient satisfaction in elective orthopedic surgeries such as arthroplasty or arthroscopic surgery [6, 8]. However, the findings in orthopedic trauma surgery remain inconsistent, particularly for distal extremity fractures. Some studies have reported unclear analgesic advantage or opioid-sparing effect [2, 11], whereas others have suggested that local anesthetic infiltration can enhance postoperative pain control and reduce opioid consumption [10, 12]. Furthermore, existing evidence is limited by heterogeneous study designs and variable methodological quality.

Although some investigations on this topic have been published, they have included mixed levels of evidence and lack quantitative analyses. Consequently, the efficacy of local anesthetic injection for pain management in distal extremity fracture surgery remains unclear. Thus, the present meta-analysis aimed to evaluate the role of peri-surgical local analgesic injections in distal radius and ankle fracture surgery in terms of postoperative pain severity and opioid consumption.

## Materials and methods

### Study strategy

This meta-analysis was conducted according to the Preferred Reporting Items for Systematic Review and Meta-Analysis (PRISMA) guidelines [13] and registered in PROSPERO (CRD420251185330). The search was conducted between July and November 2025.

A comprehensive literature search was performed in PubMed, the Cochrane Central Register of Controlled Trials, and EMBASE, without language restrictions. To ensure the inclusion of all relevant studies, no limitations were initially applied regarding study design or publication year. Database-specific search algorithms were developed, combining terms across titles, abstracts, Medical Subject Headings (MeSH), Emtree, and keywords, including “pain, postoperative” (MeSH), “fracture fixation” (MeSH), “extremities” (MeSH), “anesthesia, local” (MeSH), “analgesia” (MeSH), “injection” (tiab), and “infiltration” (tiab).

### Study selection

Two authors (YHR and MK) independently performed the initial database search and screened titles and abstracts for eligibility. Full texts were subsequently reviewed according to predefined inclusion and exclusion criteria. Any discrepancies were resolved by consensus among the reviewers. Additionally, the references of relevant articles were manually screened to identify eligible studies.

The eligibility criteria were formulated using the PICOS (Population, Intervention, Comparator, Outcome, and Study design) framework [13]. Studies were included if they met the following criteria: participants, patients with distal extremity fractures involving the distal radius and ankle; intervention, surgical fixation; comparison, local anesthetic injection or infiltration; outcome, postoperative pain; and design, randomized controlled trials. Studies were excluded if they included mixed patient populations with non-separable datasets, lacked sufficient data, or enrolled participants younger than 18 years.

### Quality assessment

The methodological quality of the included studies was assessed using the Cochrane Collaboration’s Risk of Bias Tools [13]. This tool evaluates potential biases in the following key components: random sequence generation, allocation concealment, blinding, selective reporting, and incomplete outcome data. Two reviewers (YHR and MK) independently performed the assessments, and any disagreements were resolved by consensus in consultation with a third reviewer (BHY).

### Data extraction and outcome measure

Data were extracted using a pre-designed spreadsheet, recording key information such as author names, publication year, study design, sample size, patient demographics, anesthetic methods, local injection regimens, and primary outcomes. The outcomes of interest were postoperative pain and opioid consumption, converted to intravenous morphine equivalent doses using standard equianalgesic conversion tables [11], during the early postoperative period.

### Statistical analysis

We conducted a pooled analysis to compare the clinical outcomes between the groups based on the available data. Standardized mean differences (SMD) with 95% confidence intervals (CI) were used for continuous variables and odds ratios (OR) with 95% CI were calculated for binary variables. When studies reported medians with interquartile ranges instead of means with standard deviations (SD), SDs were estimated using the quantile estimation approach [14]. Between-study heterogeneity was assessed using the I^2^ statistic, with values 25%, 50%, and 70% considered low, moderate, and high, respectively. Random-effects models were applied for moderate-to-high heterogeneity (I^2^ > 50%); otherwise, fixed-effects models were used. Statistical significance was set at *p* < 0.05.

Subgroup analyses were conducted based on the anesthetic method and presence of rebound pain to explore potential sources of heterogeneity. Sensitivity analysis was conducted by sequentially omitting individual studies to assess their impact on primary outcomes. Although a funnel plot was initially planned to evaluate publication bias, it was excluded because of limited interpretability due to the small number of included studies. Statistical analyses were conducted using Comprehensive Meta-analysis Software version 3 (Biostat, Englewood, NJ, USA) and Review Manager (version 5.3; Cochrane Collaboration, Copenhagen, Denmark).

## Results

### Characteristics and methodological quality of the included study

**Fig. 1:**
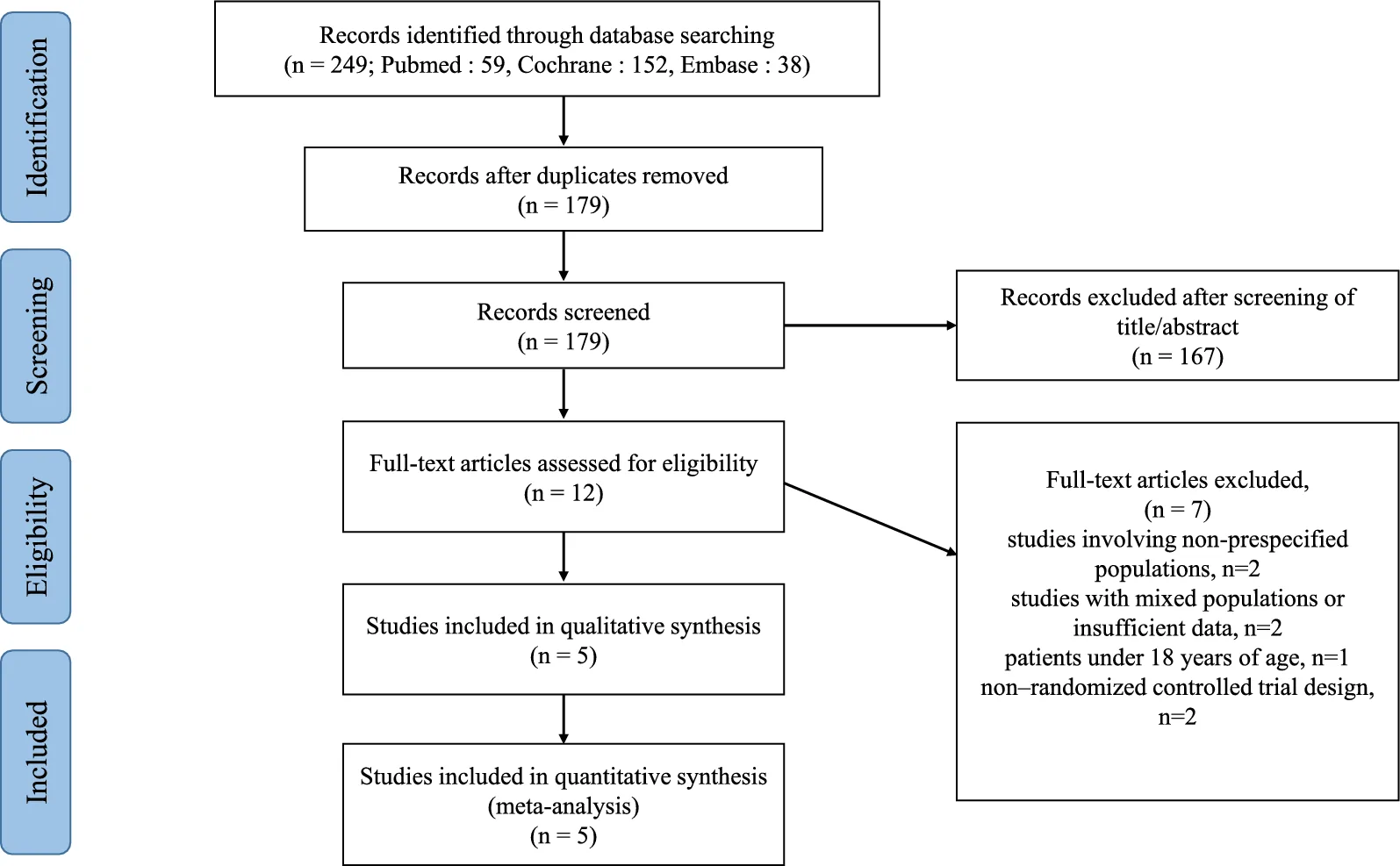
Preferred Reporting Items for Systematic Review and Meta-Analysis (PRISMA) flow diagram illustrating the study selection process

**Fig. 2:**
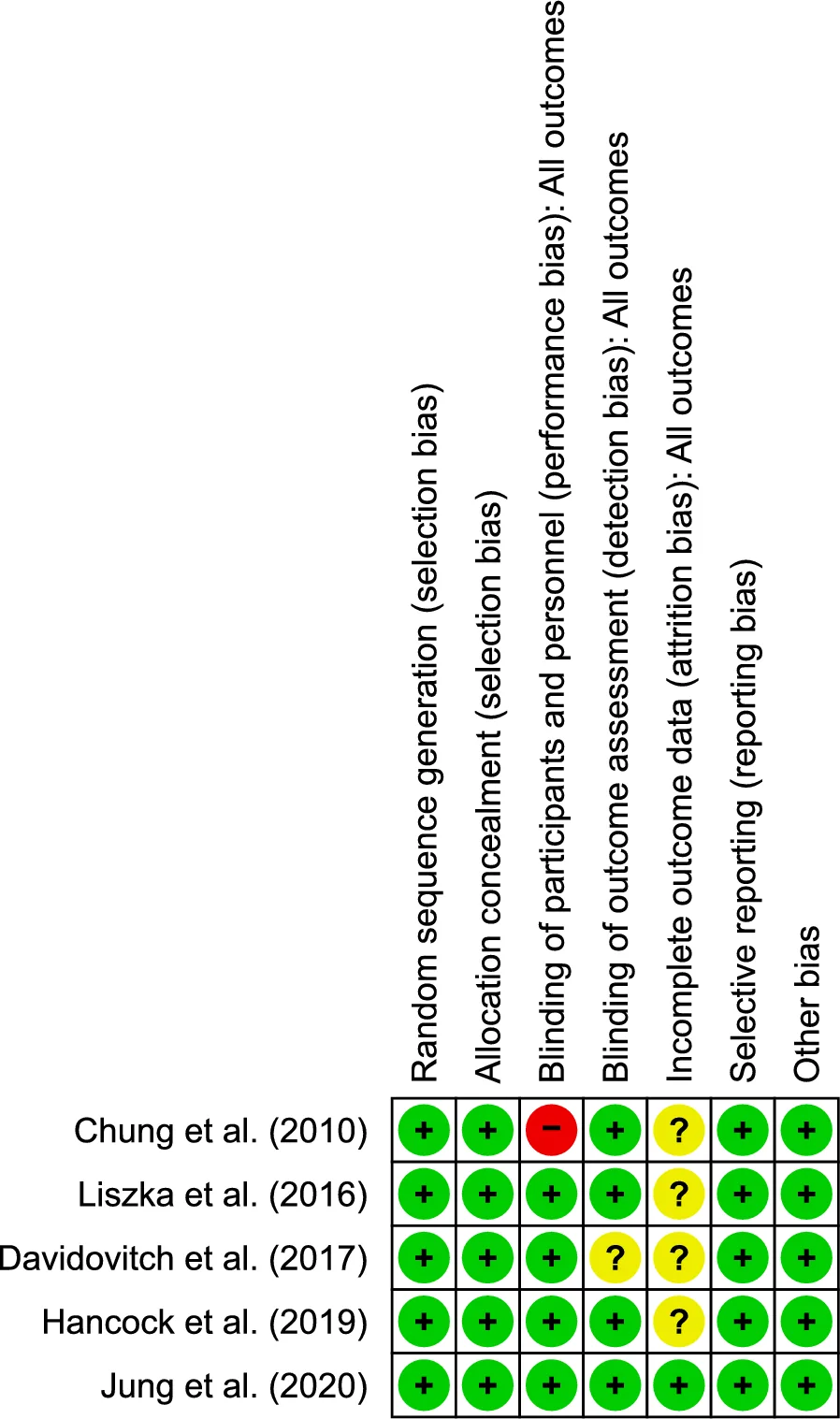
Methodological quality of the included randomized controlled trials. The risk of bias items were assessed and categorized as “low risk” ( +), “unclear risk” (?), or “high risk” (-)

A total of 249 studies were initially identified. Five studies were selected for qualitative analysis after applying the inclusion and exclusion criteria (Fig. 1). All five studies analyzed were randomized controlled trials published between 2010 and 2020, and their main characteristics are presented in Table 1. Of the included studies, three involved patients who underwent distal radius fracture surgery, while the remaining two included patients with ankle fractures. The duration of the acute postoperative pain assessment ranged from 48 to 72 h across the studies. The results of the risk of bias assessment are summarized in Fig. 2. Apart from the lack of patient blinding to the analgesic technique and the unclear reporting on the handling of incomplete data, the studies demonstrated generally acceptable methodological quality.

**Table 1:** Characteristics of the included studies

Study	Study design	Fracture site	Sample size		Mean age (yr)		Female (%)		OTA/AO classification		Anesthesia	Local analgesic solution	Postoperative evaluation (hour)
			PI	Control	PI	Control	PI	Control	PI	Control			
Chung et al. [2]	RCT	distal radius	22	22	57 ± 10	61 ± 14	73	68	A: 10 B: 1 C: 11	A: 10 B: 1 C: 11	Regional	Ropivacaine, epinephrine,morphine	4, 8, 24, 48
Liszka et al. [12]	RCT	distal radius	41	40	59 ± 30	58 ± 28	63	68	A: 0 B: 18 C: 23	A: 0 B: 19 C: 21	General	Bupivacaine, lidocaine	0, 4, 8, 12, 16, 24, 48, 72
Davidovitch et al. [10]	RCT	ankle	37	39	41.9	42.6	51	49	A: 8 B: 19 C: 10	A: 12 B: 16 C: 11	General	Liposomal bupivacaine	4, 24, 48, 72
Hancock et al. [11]	RCT	ankle	49	51	45 ± 18	44 ± 18	61	59	A: 2 B: 34 C: 13	A: 1 B: 27 C: 23	General	Ropivacaine, epinephrine,morphine	every 4 h for 48 h
Jung et al. [3]	RCT	distal radius	50	48	62 ± 13	62 ± 11	84	77	A: 14 B: 3 C: 33	A: 15 B: 2 C: 31	General	Ropivacaine, morphine	4, 8, 24, 48

RCT = randomized controlled trial, PI = perifracture site injection, AO/OTA = Arbeitsgemeinschaft für Osteosynthesefragen/Orthopaedic Trauma Association

### Outcomes

#### Postoperative pain score at 4, 8, 24, and 48 h

**Fig. 3:**
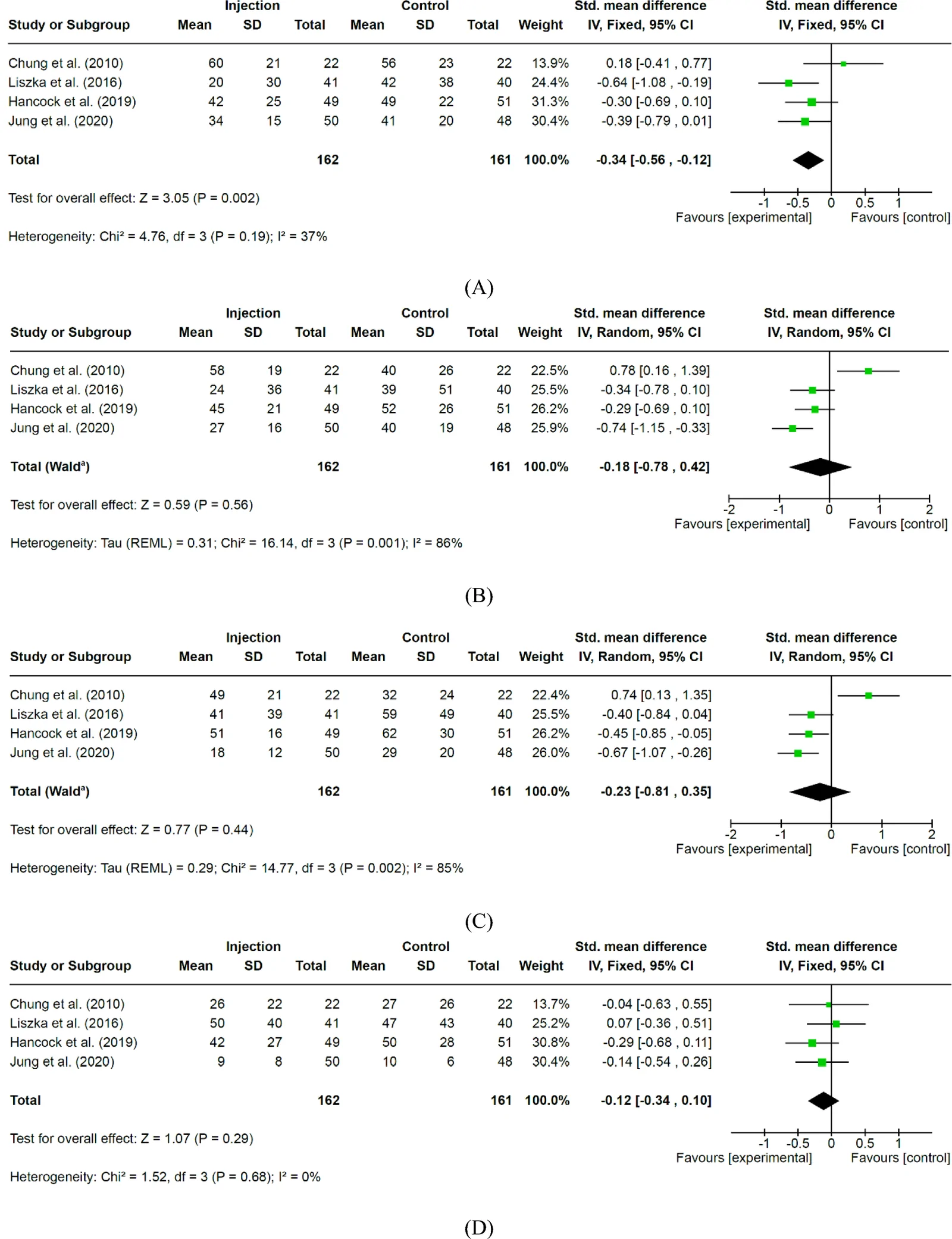
Forest plot comparing acute postoperative pain between the perifracture injection and control groups after surgical fixation of distal extremity fractures: **A** at 4 h, **B** at 8 h, **C** at 24 h, and **D** at 48 h

Postoperative pain scores assessed using visual analog scale (VAS) at 4 h were reported in four studies, including 323 patients. Among these, two studies showed significantly lower VAS pain scores in the injection group than in the control group, whereas the remaining two found no significant difference between the groups. The pooled analysis demonstrated that the injection group had significantly lower VAS scores at 4 h (standardized mean difference [SMD], − 0.34; 95% confidence interval, − 0.56 to − 0.12, I^2^ = 37%, Fig. 3A). The results of the sensitivity analysis were consistent with the primary findings.

At 8 h after surgery, four studies provided data on VAS pain scores. Of these, one study observed lower pain scores in the injection group, another showed lower pain scores in the control group, and two identified no significant differences between the groups. A pooled analysis indicated the overall difference in pain scores was not statistically significant (SMD − 0.18; 95% CI − 0.78 to 0.42, I^2^ = 86%, Fig. 3B). Sensitivity analysis, excluding the study by Chung et al., revealed significantly lower pain scores in the injection group with reduced heterogeneity (Table 2).

**Table 2:** Results of subgroup analysis for postoperative pain at 8 h and 24 h

Subgroup	No. of studies	SMD; 95% CI	Heterogeneity
Under general anesthesia, without rebound pain at 8 h	3	− 0.46; − 0.69 to − 0.22	I^2^ = 27%, *P* = 0.26
Under regional anesthesia, with rebound pain at 8 h	1	0.78; 0.16 to 1.39	NA
Under general anesthesia, without rebound pain at 24 h	3	− 0.51; − 0.75 to − 0.27	I^2^ = 0% *P* = 0.65
Under regional anesthesia, with rebound pain at 24 h	1	0.74; 0.13 to 1.35	NA

SMD, standardized mean difference; CI, confidence interval

At 24 h postoperatively, the results were comparable with those observed at 8 h. One study reported lower pain scores in the injection group, another favored the control group, and the remaining two studies found no significant differences between the groups. The pooled analysis indicated no significant difference in pain scores between groups (SMD − 0.23; 95% CI − 0.81 to 0.35, I^2^ = 85%, Fig. 3C). However, after excluding the study by Chung et al. in the sensitivity analysis, the injection group exhibited lower pain scores accompanied by low heterogeneity (Table 2).

At 48 h, all four studies consistently reported no significant differences in VAS scores for postoperative pain between the groups. The results of the meta-analysis confirmed the absence of a statistically significant difference in postoperative pain at 48 h (Fig. 3D; SMD − 0.12; 95% CI − 0.34 to 0.10; I^2^ = 0%). The findings of the sensitivity analysis were consistent with those of the primary analysis.

#### Amount of opioid consumption

**Fig. 4:**
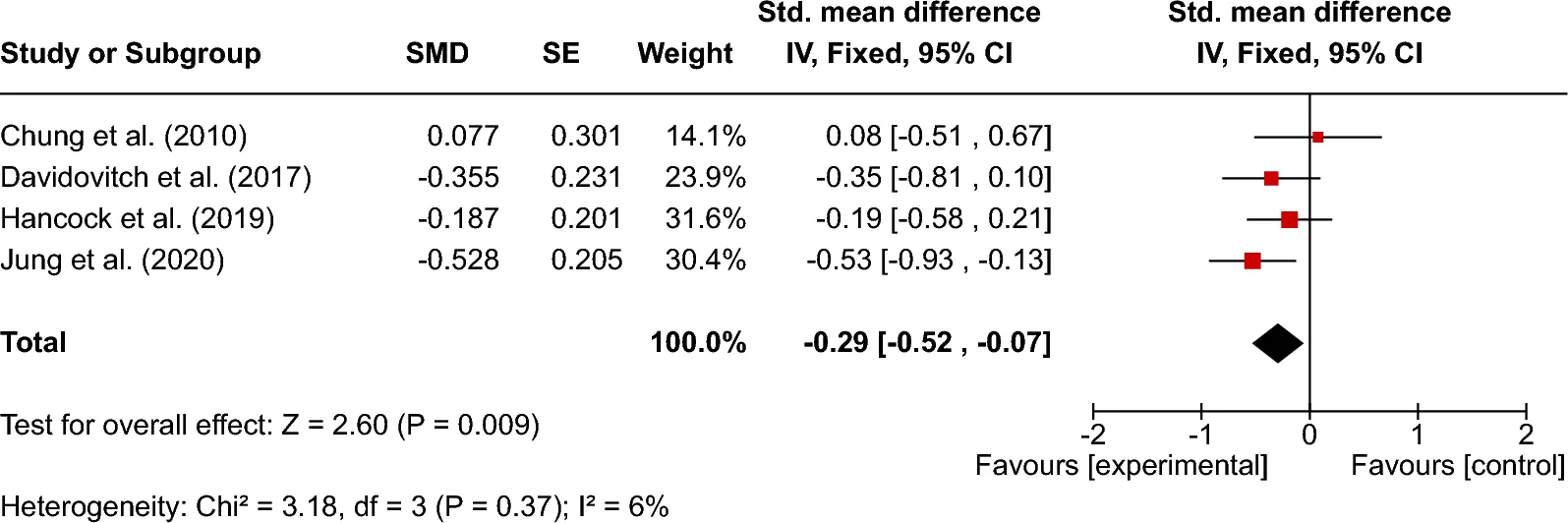
Forest plot comparing total opioid consumption between the perifracture injection and control groups following surgical fixation of distal extremity fractures

Four studies, comprising 318 extremities, assessed opioid consumption following the fracture surgery. Of these, one study found lower opioid use in the injection group than in the control group, whereas the remaining three studies reported no statistically significant differences between the groups. The results of the meta-analysis demonstrated that the injection group had a significantly lower total opioid consumption than the control group (Fig. 4, SMD − 0.29; 95% CI − 0.52 to − 0.07; I^2^ = 6%). The results of the sensitivity analysis were consistent with the primary findings.

## Discussion

Several studies have evaluated the efficacy of perifracture site injections for postoperative pain management; however, their findings have been inconsistent. In the present meta-analysis, which included five randomized controlled trials, perifracture site injections demonstrated significant analgesic efficacy confined to the early postoperative period, accompanied by an overall reduction in opioid consumption. Substantial heterogeneity was observed on the first postoperative day, which was potentially attributable to rebound pain.

Perifracture site injection was associated with significantly lower postoperative pain scores at 4 h compared to the control group, with low between-study heterogeneity. However, no significant differences in pain scores were observed at 8 h or 24 h, and substantial heterogeneity was observed across the studies. One study using regional anesthesia reported that patients who received perifracture site injections experienced a rapid increase in pain scores on the first postoperative day, likely after the local anesthetic effect had worn off [2]. This finding suggests that the heterogeneity in pain scores between 8 and 24 h may have been influenced by the incidence or severity of rebound pain [15]. Sensitivity analysis, excluding this study, demonstrated lower pain scores and reduced heterogeneity among patients treated with perifracture site injections. These results indicate that effective management of rebound pain is essential for prolonging the analgesic benefits of local injections. Nonetheless, the magnitude of pain reduction observed in these studies was small and unlikely to be clinically meaningful, with no corresponding improvement in patient satisfaction [3, 10]. Although the pooled analysis demonstrated statistical significance, the between-group difference of postoperative pain at 4 h was approximately 9.5 points on a 100-point VAS scale, which is close to but slightly below the reported minimal clinically important difference (MCID) of 9.9 points for acute postoperative pain [16]. Therefore, the current evidence provides limited support for perifracture site injection in distal extremity fractures as a strategy for sustained postoperative pain relief.

The total opioid consumption after distal radius and ankle fracture surgery was reduced in patients who received perifracture site injections, although this effect was less pronounced than that observed in arthroplasty or hip fracture procedures. Two studies showed a trend toward reduced opioid use in patients receiving perifracture injections; however, the differences were not statistically significant [10, 11]. Reducing postoperative opioid requirements remains an important goal of multimodal pain management, given the well-recognized adverse effects of opioids and concerns about over-prescription [17, 18]. Although published findings regarding the effect of local injections on opioid consumption after distal extremity fracture surgery are inconsistent, our meta-analysis indicated an overall reduction in total opioid use during the early postoperative period.

The limited analgesic efficacy of local injection in distal extremity fractures compared with findings reported for arthroplasty or hip fracture surgery may contribute to differences in surgical invasiveness and tissue characteristics of the involved joint. Open reduction and fixation for distal extremity fractures generally requires less extensive dissection and shorter operative times than arthroplasty or hip fracture procedures [3]. In addition, differences in the soft tissue envelope surrounding the distal extremity joints may influence the distribution and absorption of locally injected analgesics [11]. Previous studies assessing multimodal periarticular injections after hip or knee arthroplasty have reported mean pain scores of approximately 60 at 24 h postoperatively [19, 20], with a longer duration of intense pain compared with distal extremity fractures [2]. Nevertheless, a substantial proportion of patients with distal extremity fractures experience moderate-to-severe postoperative pain [21, 22]. Therefore, there is a need for more effective analgesic strategies for these patients, potentially through local injections in combination with a multimodal analgesic regimen, where synergistic effects may provide clinically meaningful pain relief.

Our study has some limitations. First, the number of studies included was limited. Because the inclusion criteria were restricted to randomized controlled trials, the pool of eligible studies for the meta-analysis was relatively small. However, this approach was chosen to ensure a high level of evidence and to support high-grade recommendations. Second, differences in anesthetic techniques, including general versus regional anesthesia, may have influenced the observed outcomes, introducing clinical heterogeneity across the included studies. To address this issue, we performed additional secondary analyses according to anesthetic type at time points demonstrating significant heterogeneity. Third, the protocol for perifracture site injection has not been standardized and varied across studies. In addition, differences in postoperative pain management protocols among the included trials may have contributed to heterogeneity. Although these variations reflect real-world clinical practice, they are potential sources of bias. Fourth, heterogeneity was also observed in reporting of outcomes, as the timing and methods of follow-up assessments differed among the studies. Further research with larger sample sizes and diverse study settings is needed to clarify these findings and strengthen the evidence base.

## Conclusions

Perifracture site injections for distal radius and ankle fractures provided significant analgesic efficacy confined to the early postoperative period, along with an overall reduction in opioid consumption. However, this effect appeared less pronounced than that observed during arthroplasty or hip fracture procedures. The substantial variability in pain severity on the first operative day may be partly attributed to rebound pain. Further investigations are needed to clarify the optimal injection protocol and determine whether combining this approach with multimodal analgesia can yield more sustained postoperative pain relief.

## Data Availability

All data supporting the findings of this study were extracted from previously published randomized controlled trials, which are cited in the manuscript. No new experimental or clinical data were generated. The data presented in this study are available upon request to the corresponding author.
